# Anti-Inflammatory Effects of *Chamaecyparis obtusa* (Siebold & Zucc.) Endl. Leaf Extract Fermented by *Ganoderma applanatum* Mycelia

**DOI:** 10.3390/pharmaceutics16030365

**Published:** 2024-03-05

**Authors:** Chae-Hyun Kim, Yong-Jin Kwon, Young-Ah Jang

**Affiliations:** 1 Department of Cosmeceutical Science, Kyungsung University, Busan 48434, Republic of Korea; cogus198@naver.com; 2 Department of Cosmetic Science, Kyungsung University, Busan 48434, Republic of Korea; 3 Division of Cosmetic Science, Daegu Hanny University, Gyeongsan-si 38610, Republic of Korea

**Keywords:** *Chamaecyparis obtusa* (Siebold & Zucc.) endl., *Ganoderma applanatum*, anti-inflammation, macrophage, STAT, pro-inflammatory molecules

## Abstract

Corticosteroids are commonly used anti-inflammatory agents. However, their prolonged use can lead to side effects. Therefore, the development of natural compounds with minimal side effects is necessary. This study was performed to investigate the anti-inflammatory effects and mechanisms of action of *Chamaecyparis obtusa* (Siebold & Zucc.) Endl. leaf (COL), bioconverted using *Ganoderma applanatum* (*G. applanatum*) in lipopolysaccharide (LPS)-induced RAW264.7 cells. The COL 70% EtOH extract fermented by *G. applanatum* (70COLGA) improved the high cytotoxicity of 70% EtOH extracts (70COL). When RAW264.7 cells were pre-treated with 100 and 200 μg/mL of 70COLGA for 2 h and then treated with LPS for 16 h, LPS induced the production of nitric oxide (NO), and the expressions of inducible nitric oxide synthase (iNOS) and cyclooxygenase 2 (COX-2) were significantly inhibited. When RAW264.7 cells were pre-treated with 100 and 200 μg/mL of 70COLGA for 2 h and then treated with LPS for 4 h, the phosphorylation of signal transducers and activators of transcription (STAT) was markedly decreased. In addition, 70COLGA markedly suppressed the production of the inflammatory cytokines interleukin (IL)-1β and IL-6 in LPS-induced RAW264.7 cells. Analysis of pro-inflammatory molecules using cytokine arrays showed that macrophage inflammatory protein (MIP)-2, granulocyte–macrophage colony-stimulating factor (GM-CSF), granulocyte colony-stimulating factor (G-CSF) and IL-27 expressions were also suppressed by 200 μg/mL of 70COLGA in LPS-induced RAW264.7 cells. These results demonstrate that 70COLGA significantly prevented inflammatory responses by inhibiting the secretion of pro-inflammatory molecules in LPS-induced RAW264.7 cells. When RAW264.7 cells were pre-treated with 100 and 200 μg/mL of 70COLGA for 2 h and then treated with LPS-conditioned medium (LPS-CM) for 30 min, 70COLGA directly inhibited STAT activation. In summary, our findings suggest that 70COLGA has therapeutic potential for the treatment of inflammatory diseases.

## 1. Introduction

Inflammatory responses are a defense mechanism of the body against external stimuli or the invasion of pathogens [1]. Inflammatory responses are initiated when pathogen-associated molecular patterns (PAMPs) derived from pathogens are recognized by tissue-resident leukocytes [2]. These white blood cells release cytokines or chemokines that promote the migration of immune cells to the site of inflammation [3]. Migrated immune cells remove pathogens and dead cells from the site of infection and repair damaged tissue [4]. Thus, an appropriate inflammatory response is necessary for the restoration of homeostasis in damaged tissues [5]. However, excessive and prolonged inflammatory responses can lead to chronic inflammatory skin diseases, such as atopic dermatitis, seborrheic dermatitis, and psoriasis [6,7]. Therefore, it is important to devise treatment strategies to inhibit excessive inflammatory responses.

Macrophages are white blood cells that play important roles in regulating inflammatory responses [8]. Lipopolysaccharide (LPS) is an important outer membrane component of Gram-negative bacteria, and is commonly used in vitro to stimulate macrophages for an evaluation of anti-inflammatory agents [9]. Prolonged inflammation with excessive production of inflammatory mediators and cytokines induced by external stimuli can lead to cellular and tissue damage [1]. Currently, topical corticosteroids are widely used in the treatment of chronic inflammatory skin diseases, but their long-term use causes side effects, such as atopic dermatitis, contact allergies, and acne [10,11]. Therefore, there is a great deal of interest in the development of natural compounds with safe anti-inflammatory effects and minimal side effects.

*Chamaecyparis obtusa* (Siebold & Zucc.) Endl. (CO) belongs to the Cupressaceae family and is currently widely cultivated in Japan and Southern Korea. CO has been used as a traditional medicine to prevent allergies and relieve inflammation in East Asian countries, and is reported to have anti-inflammatory, antioxidant, and anti-cancer effects [12,13,14,15]. CO essential oil, the main ingredients of which are monoterpenes and sesquiterpenes, is known to relieve itching of atopic dermatitis and to have an anti-bacterial effect on skin microorganisms [16]. Although CO extract is already well known for its anti-inflammatory effects, we aim to develop new materials that are more effective in treating inflammation using CO.

Bioconversion is a technique that uses the enzymatic functions of microorganisms to increase the physiological activity of materials or reduce toxicity [17]. Recent research has focused on improving the physiological activities of materials through fermentation using microorganisms such as lactic acid bacteria and fungi [18,19]. It has been reported that materials converted through bioconversion technology have improved antioxidant and anti-inflammatory effects compared to existing materials [20,21]. Previous studies have shown that *Ganoderma lucidum*, a fungus belonging to the Ganodermataceae, enhanced anti-inflammatory effects in a model of atopic dermatitis through bioconversion [22]. Several studies have shown that *Ganoderma applanatum* (*G. applanatum*) has anti-bacterial, anti-tumor, anti-viral, and antioxidant properties, but there have been no reports regarding bioconversion by inoculating CO with *G. applanatum* mycelia (GAM) [23]. Therefore, this study was performed to examine the anti-inflammatory effects of an extract obtained by bioconversion by inoculating CO with GAM using RAW264.7 cells, a mouse-derived macrophage cell line commonly used for anti-inflammatory evaluations [24].

## 2. Materials and Methods

### 2.1. Reagents Used in This Study

2,2-Diphenyl-1-picrylhydrazyl (DPPH, D9132), 2,2′-Azino-bis(3-ethylbenzothiazoline-6-sulfonic acid) diammonium salt (ABTS, 11557), potassium persulfate (216224), L-ascorbic acid (A0278), tannic acid (403040), quercetin (Q4591), lipopolysaccharide (LPS, L3012), butylated hydroxyanisole (BHA, B1253) and Griess reagent (G4410) were purchased from Sigma Aldrich (St. Louis, MO, USA). Phenol reagent (Folin–Ciocalteu’s reagent, 96703S8130) and sodium hydroxide (2020K1192) were purchased from Junsei Chemical Co., Ltd. (Tokyo, Japan). Sodium carbonate anhydrous (7541–4405) was purchased from Daejung Chemicals & Metals Co., Ltd. (Siheung-si, Gyeonggi-do, Republic of Korea). Diethylene glycol was purchased from Samchun Chemical (Seoul, Republic of Korea). 3-(4,5-dimethylthiazol-2-Yl)-2,5-diphenyltetrazolium bromide (MTT) reagent (M1415) was purchased from Duchefa Biochemie (Haarlem, The Netherlands). These reagents’ characteristics are summarized in Table S1.

### 2.2. Antibodies Used in This Study

Anti-COX-2 (#12282), anti-pY-STAT1 (#8826), anti-STAT1 (#9172), anti-pY-STAT3 (#9145), anti-STAT3 (#30835), anti-pT/Y-p44/42 MAPK (Erk1/2) (#9101), anti-p44/42 MAPK (Erk1/2) (#4695), anti-pT/Y-SAPK/JNK (#9251), anti-SAPK/JNK (#9252), anti-pT/Y-p38 MAPK (#9211), anti-p38 MAPK (#9212), anti-pS-IκBα (#2859) and anti-pS-NFκB (#3033) were purchased from Cell Signaling Technology (Danvers, MA, USA). Anti-IκBα (sc-1643), anti-NFκB (sc-8008) and anti-β-Actin (sc-47778) were purchased from Santa Cruz Biotechnology (Santa Cruz, CA, USA). The horseradish peroxidase (HRP)-tagged anti-rabbit antibody (A21010) was purchased from Abbkine (Wuhan, China). The HRP-tagged anti-mouse (ADI-SAB-100) was purchased from Enzo Life Science (Farmingdale, NY, USA). These antibodies’ characteristics are summarized in Table S2.

### 2.3. Extraction of 70COL and 70COLGA 

The CO leaves (COLs) used in this study were collected from Chukdong-myeon, Sacheon-si, Gyeongsangnam-do, Korea, in May 2022. A 70% EtOH extract of COLs (70COL) was prepared by extraction of COLs twice with a 10-fold excess (*w*/*v*) of 70% EtOH for 24 h each time at room temperature. The 70COL was passed through a paper filter (Whatman™ Quantitative Filter Paper; Toyo Kaisha, Tokyo, Japan) and then concentrated at 50 rpm and 40 °C using a vacuum rotary evaporator (HS-10SP; Hahnshin S&T, Gimpo, Republic of Korea). The concentrated extract was freeze-dried using a freeze dryer (Freeze Dryer with Micro Concentrator MCFD; ilShin Biobase, Dongducheon-si, Gyeonggi-do, Republic of Korea). To prepare a 70% EtOH extract (70COLGA) inoculated with mycelium, 10% of GAM cultured in a PDB medium for 14 days was inoculated into a concentrated 70COL solution using a vacuum rotary evaporator, and fermentation was carried out at 180 rpm and 25 °C for 3 weeks. The extract was passed through a paper filter (Toyo Kaisha) and then freeze-dried using a freeze dryer (ilShin Biobase). The extracts were stored at 4 °C before use.

### 2.4. High-Performance Liquid Chromatography (HPLC) Fingerprint Analysis

The sample solution was prepared by dissolving 10 mg of extracts in 1 mL of MeOH solvent. Thereafter, the supernatant was filtered through a 0.22 µm syringe filter. A chemical fingerprint analysis of extracts was carried out using an Agilent HPLC 1260 (Thermo Fisher Scientific, Waltham, MA, USA). Chromatographic separation was performed using an Agilent Eclipse XDB-C18 (4.6 × 250 mm, 5 µm) analytical column. The HPLC conditions are summarized in Table S3.

### 2.5. Total Polyphenolic Compound Content Assay

A total of 50 µL of extracts was mixed with 50 µL of Folin–Ciocalteu reagent. After 5 min at room temperature and after being kept in the dark, 50 µL of 10% Na_2_CO_3_ was added. The reaction mixture was incubated at room temperature for 1 h. The absorbance was measured at 640 nm using a plate reader (BioTek, Winooski, VT, USA) and the data were expressed as milligrams of tannic acid equivalent per gram of sample (mg TAE/g).

### 2.6. Total Flavonoid Compound Content Assay

The extract, diethylene glycol, and sodium hydroxide were mixed in a ratio of 1:10:10. After reacting at 37 °C for 1 h, the absorbance was measured at 420 nm using a plate reader (BioTek). Data are expressed as milligrams of quercetin equivalent per gram of sample (mg QE/g).

### 2.7. DPPH Radical Scavenging Activity Assay

An aliquot of 50 µL of 0.2 mM DPPH solution in 99% EtOH was mixed with 100 µL of extract at concentrations of 50, 100, 200, and 400 µg/mL. The mixture was then incubated at room temperature for 30 min in the dark. The absorbance was measured at 517 nm using a plate reader (BioTek). Butylated hydroxyanisole (BHA) was used as a positive control.

### 2.8. ABTS^+^ Radical Scavenging Activity Assay

After mixing 7.4 mM ABTS and 2.6 mM potassium persulfate in a 1:1 ratio, the solution was allowed to react for 24 h at room temperature in the dark. Before use, the ABTS^+^ solution was diluted with 99% EtOH to obtain an absorbance value of approximately 0.7. The diluted ABTS^+^ solution and extracts at concentrations of 50, 100, 200, and 400 µg/mL were mixed in a 1:1 ratio and allowed to react for 1 min at room temperature in the dark. The absorbance was measured at 734 nm using a plate reader (BioTek). Ascorbic acid (AA) was used as a positive control.

### 2.9. Cell Line and Culture

The mouse macrophage cell line RAW264.7 was purchased from the American Type Culture Collection (ATCC) (Rockville, MD, USA). The cells were cultured in Dulbecco’s Modified Eagle Medium (DMEM, Capricorn Scientific GmbH, Ebsdorfergrund, Germany) containing 10% heat-inactivated fetal bovine serum (FBS, Capricorn Scientific GmbH) and 1% penicillin/streptomycin (Capricorn Scientific GmbH). The cells were incubated in a humidified incubator (PHCbi CO_2_ Incubator, Tokyo, Japan) in 5% CO_2_ at 37 °C. Cells were subcultured once every two to three days.

### 2.10. Cell Viability Assay

Cell viability was determined via an MTT assay. RAW264.7 cells were seeded in 96-well culture plates and treated with extracts at concentrations of 25, 50, 100, 200, 400, and 800 µg/mL for 24 h. After treatment, cells were treated with MTT reagent (5 mg/mL) and incubated for 2 h. The formazan crystals were dissolved in 100 µL of DMSO and the absorbance was determined at 570 nm using a plate reader (BioTek).

### 2.11. Nitric Oxide (NO) Assay

NO levels in cell culture media supernatant were determined using the Griess reagent. RAW264.7 cells were seeded in 6-well culture plates and incubated overnight. After pre-treatment with extracts at concentrations of 100 and 200 µg/mL for 2 h, LPS (200 ng/mL) was treated for 16 h. To measure NO production, 100 µL of supernatant was reacted with 100 µL of Griess reagent for 10 min in the dark. The absorbance was measured at 540 nm using a plate reader (BioTek).

### 2.12. Western Blot

Cells were washed with cold phosphate-buffered saline (PBS) and proteins were extracted using 0.5% Triton X-100 buffer (Sigma Aldrich) containing protease and phosphatase inhibitors (2 mM phenylmethanesulfonyl fluoride, PMSF; 1 mM sodium fluoride, NaF; 2 mM ethylenediaminetetraacetic acid, EDTA; 0.5 mM sodium orthovanadate, Na_3_VO_4_; 10 μg/mL leupeptin). After incubation for 10 min on ice, lysates were centrifuged at 13,000 rpm for 10 min at 4 °C. The supernatant was collected. Lysates were separated on SDS-polyacrylamide gels and transferred to nitrocellulose (NC) membranes. The membranes were blocked in 5% skim milk for 1 h at room temperature and then incubated with specific primary antibodies (diluted to 1:1000) at 4 °C overnight. The next day, membranes were incubated with HRP-conjugated secondary antibodies at room temperature for 1 h. The membrane was incubated with ECL solution (Bio-Rad, Hercules, CA, USA) for 1 min. Images were obtained using a Davinch-Chemi Imager™ CAS-400SM (Davinch-K, Seoul, Republic of Korea).

### 2.13. RNA Isolation and Quantitative Real-Time PCR (qPCR)

Total RNA was extracted using RNAiso Plus reagent (Takara, Kusatsu, Shiga, Japan), and cDNA was synthesized using a ReverTra Ace qPCR RT Master Mix (TOYOBO, Osaka, Japan) according to the manufacturer’s instructions. qPCR was performed using the SYBR Green qPCR Master Mix (Applied Biological Materials, Richmond, BC, Canada) and amplification was achieved using a LightCycler^®^96 System (Roche, Basel, Switzerland). The PCR conditions included denaturation at 95 °C for 15 s and annealing/extension at 60 °C for 1 min, repeated 40 times. Relative gene expression levels were normalized to GAPDH expression levels. The sequences of qPCR primers are summarized in Table 1.

### 2.14. Cytokine Array

RAW264.7 cells were pretreated with extract for 2 h, and LPS (200 ng/mL) was added for 4 h. Culture supernatants were collected, and cytokines were measured using the Mouse Cytokine Array Panel kit according to the manufacturer’s instructions (ARY006; R&D systems, Minneapolis, MN, USA). Images were obtained using a Davinch-Chemi Imager™ CAS-400SM (Davinch-K) and the blots of cytokine were quantified using Image J (bundled with 64-bit Java 8).

### 2.15. Conditioned Medium (CM) from LPS-Induced Macrophages and Heat-Inactivated CM

RAW264.7 cells were activated by treatment with LPS (200 ng/mL) for 4 h. After washing the LPS-treated medium with PBS, it was replaced with a new medium and cultured for another 4 h. Heat-inactivated CM was synthesized by boiling LPS-CM at 100 °C for 20 min to degrade all secreted cytokines and chemokines, and was used as a negative control.

### 2.16. Dataset Analysis

The dataset used in this study is available at the Gene Expression Omnibus (GEO) database (http://www.ncbi.nlm.nih.gov/geo/ (accessed on 5 December 2023)) under the accession number GSE76563. The Z-score for each gene of interest was calculated and displayed using a heatmap using Microsoft Excel software (Microsoft office 365, Version 2420, Redmond, WA, USA).

### 2.17. Statistical Analysis

Statistical analysis of all data was performed using Microsoft Excel software (Microsoft office 365, Version 2420). All experiments were repeated at least three times independently. Significance was considered at *p*-values < 0.05.

## 3. Results

### 3.1. HPLC Analysis and Antioxidant Activity of the 70COL and 70COLGA

**Figure 1:**
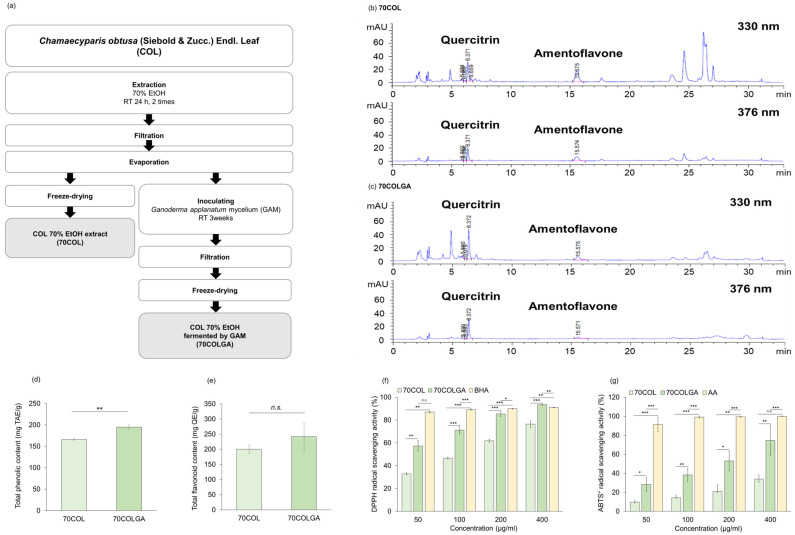
Antioxidant activity of 70COL and 70COLGA. (**a**) Overview of the extraction method of 70COL and 70COLGA. (**b**,**c**) Representative HPLC chromatograms of 70COL (**b**) and 70COLGA (**c**). (**d**) Total phenolic content of 70COL and 70COLGA. (**e**) Total flavonoid content of 70COL and 70COLGA. (**f**) DPPH radical scavenging activity of 70COL and 70COLGA. (**g**) ABTS^+^ radical scavenging activity of 70COL and 70COLGA. All data represent the mean ± SD *(n* = 3). *n.s*.: not significant, * *p* < 0.05, ** *p* < 0.01 and *** *p* < 0.001 by Student’s *t*-test.

This study was performed to compare and evaluate the anti-inflammatory effects of 70COL and 70COLGA. First, 70COL was extracted by immersing COL in 70% EtOH, and 70COLGA was extracted by immersing COL in 70% EtOH and inoculating with GAM (Figure 1a). The major flavonoids of CO were reported to be quercitrin, amentoflavone, and myricetin [25,26]. HPLC fingerprint analysis revealed that 70COL had a high amentoflavone content (70COL: 26.66%, 70COLGA: 8.84%), while 70COLGA had a high quercitrin content (70COL: 15.08%, 70COLGA: 20.52%), indicating that the composition changed through bioconversion (Figure 1b,c, Tables S4 and S5). The total phenol and flavonoid contents were confirmed as the flavonoid contents changed. 70COLGA had a higher total phenolic and flavonoid content than 70COL (Figure 1d,e). As polyphenols are closely related to antioxidant activity, a DPPH assay and an ABTS^+^ assay were performed to confirm antioxidant activity (Figure 1f,g) [27]. The results showed that 70COLGA had a higher antioxidant activity than 70COL at low concentrations. Antioxidant activity is associated with anti-inflammatory effects, so it was expected that 70COLGA, which had a higher antioxidant effect at low concentrations, would have a greater anti-inflammatory effect [28].

### 3.2. 70COLGA Suppresses LPS-Induced Inflammation in RAW264.7 Cells

**Figure 2:**
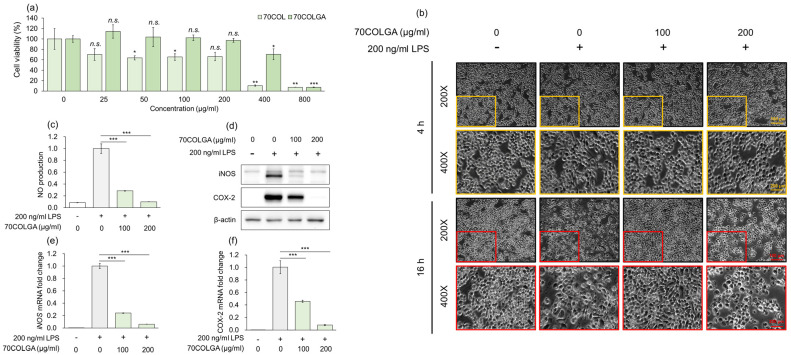
70COLGA had high anti-inflammatory efficacy. (**a**) RAW264.7 cells were treated with 70COL and 70COLGA at the indicated concentrations (25, 50, 100, 200, 400 and 800 μg/mL) for 24 h. Cell viability was determined using the MTT assay. (**b**–**f**) RAW 264.7 cells were pre-treated with 70COLGA at 100 and 200 μg/mL concentration for 2 h and then incubated with LPS for 16 h. Morphological changes to RAW264.7 cells are shown. Scale bar: 100, 200 μm (**b**). The production of NO was determined using Griess reagent (**c**). The relative protein levels were analyzed via a Western blot analysis (**d**). The relative mRNA expression was analyzed via RT-qPCR (**e**,**f**). Data are expressed as the mean ± SD, *n* = 3. *n.s*.: not significant, * *p* < 0.05, ** *p* < 0.01, *** *p* < 0.001 vs. LPS-treated alone by Student’s *t*-test.

An MTT assay was performed to assess the toxicity of 70COL and 70COLGA prior to the experiments. While 70COL exhibited a cell viability of <80% at a concentration of 25 µg/mL, 70COLGA showed nearly 100% cell viability even at concentrations up to 200 µg/mL (Figure 2a). Therefore, it was confirmed that bioconversion to 70COLGA reduced the cytotoxicity of 70COL. As a cell viability < 80% is considered indicative of cytotoxicity, 70COL was excluded from subsequent experiments [29]. To confirm the anti-inflammatory effect of 70COLGA, RAW264.7 cells were stimulated with LPS, which is known to induce morphological changes in these cells [30]. We found that 70COGLA inhibited the LPS-induced morphological changes to RAW264.7 cells in a concentration-dependent manner (Figure 2b). In addition, 70COLGA significantly reduced LPS-induced inducible nitric oxide synthase (iNOS) and cyclooxygenase 2 (COX-2) levels, mRNA and protein expressions and nitric oxide (NO) production (Figure 2c–f). Taken together, these observations confirm that 70COLGA has an anti-inflammatory effect.

### 3.3. 70COLGA Inhibits Pro-Inflammatory Cytokine Production and STAT Activation in LPS-Induced RAW264.7 Cells 

**Figure 3:**
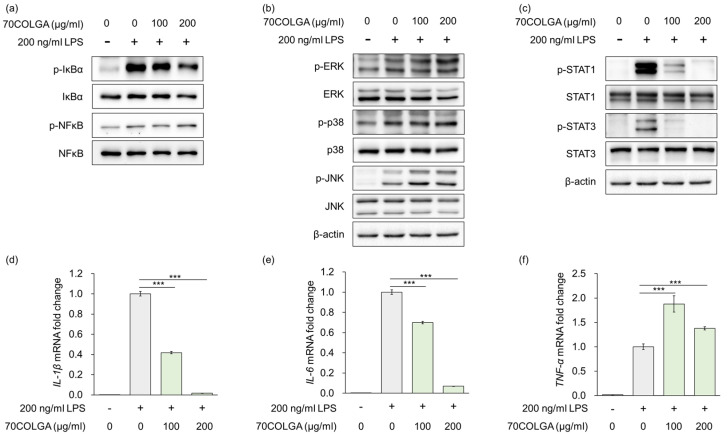
70COLGA inhibits IL production and STAT activation in LPS-induced RAW264.7 cells. (**a**–**f**). RAW 264.7 cells were pre-treated with 70COLGA at 100 and 200 μg/mL concentration for 2 h and then incubated with LPS for 4 h. The relative protein levels were analyzed via a Western blot analysis (**a**–**c**). The relative mRNA expression was analyzed via RT-qPCR (**d**–**f**). Data are expressed as the mean ± SD, *n* = 3. *** *p* < 0.001 vs. LPS-treated alone by Student’s *t*-test.

Stimulation of macrophages by LPS is known to regulate the expression of inflammatory mediators and pro-inflammatory cytokines via mitogen-activated protein kinase (MAPK), nuclear factor-kappa B (NF-κB)/NF-kappa-B inhibitor alpha (IκBα) and Janus kinase-signal transducer and activator transcription factor (JAK/STAT) signaling [31,32]. As 70COLGA suppressed LPS-induced inflammation, the MAPK and NF-κB/IκBα signaling pathways were investigated to determine the anti-inflammatory mechanism of action of 70COLGA. The results showed that 70COGLA did not prevent activation of LPS-induced MAPK and NF-κB/IκBα signaling (Figure 3a,b). However, 70COLGA significantly inhibited the activation of STAT1 and 3 (Figure 3c). When macrophages are activated by harmful substances, they secrete inflammatory cytokines during phagocytosis, including interleukin (IL)-1β, IL-6, and tumor necrosis factor-α (TNF-α) [33]. To investigate the anti-inflammatory effects of 70COLGA further, we examined LPS-induced IL-1β, IL-6, and TNF-α mRNA expressions. 70COLGA inhibited LPS-induced mRNA expression of IL-1β and IL-6, but not that of TNF-α (Figure 3d–e). These observations suggest that 70COLGA inhibits LPS-induced STAT activation and expression of the pro-inflammatory cytokines IL-1β and IL-6.

### 3.4. 70COLGA Suppresses Pro-Inflammatory Molecules in LPS-Induced RAW264.7 Cells

**Figure 4:**
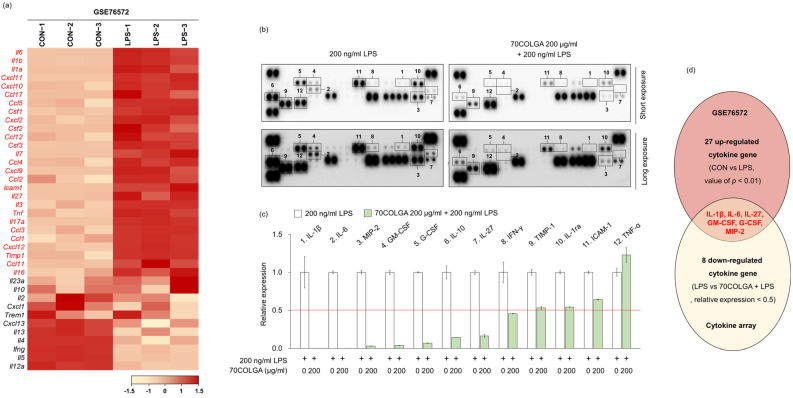
70COLGA suppresses LPS-induced pro-inflammatory molecules. (**a**) The heatmap visualization of mRNA expression differences in control (CON) and LPS-treated groups of RAW264.7 cells, based on the GSE76572 dataset. Each gene was calculated by Z-scores. (**b**,**c**) RAW 264.7 cells were pre-treated with 70COLGA at 200 μg/mL concentration for 2 h and then incubated with LPS for 4 h. The cytokines in supernatants were measured using a cytokine array kit (**b**). The blots of cytokine were quantified using Image J. The red line indicates a relative decrease in expression of more than half compared to the 200 ng/mL LPS-treated group.(**c**). (**d**) The Venn diagram illustration shows 27 cytokines genes upregulated in the GSE6572 dataset and 8 cytokines downregulated in the cytokine array data (LPS vs. 70COLGA + LPS).

Next, we investigated whether 70COLGA showed anti-inflammatory effects through inhibition of other pro-inflammatory molecules in addition to IL-1β and IL-6. First, 27 genes upregulated in LPS-induced RAW264.7 cells were identified using the GSE76562 database (Figure 4a). Cytokine array analyses showed that IL-27, macrophage inflammatory protein (MIP)-2, granulocyte-macrophage colony-stimulating factor (GM-CSF) and granulocyte colony-stimulating factor (G-CSF) expressions were increased by LPS and decreased by 70COLGA (Figure 4b–d). GM-CSF is well known as a pro-inflammatory cytokine, and G-CSF regulates the cytokine response by inducing increases in the number and activation of immune cells [34,35]. In addition, IL-27 binds to the IL-27 receptor and activates JAK-STAT signaling in macrophages, and MIP-2 has been reported to be upregulated in the skin of patients with psoriasis, a chronic inflammatory skin disease [36,37]. Therefore, 70COLGA was suggested to exhibit anti-inflammatory effects by inhibiting pro-inflammatory molecules, such as IL-27, MIP-2, GM-CSF, and G-CSF, as well as IL-1β and IL-6.

### 3.5. 70COLGA Directly Inhibited STAT Activation in RAW264.7 Cells

**Figure 5:**
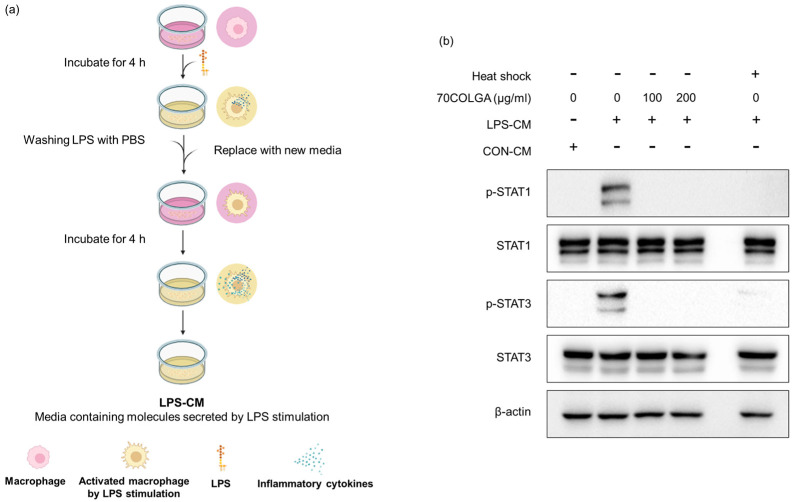
70COLGA directly inhibits STAT activation in RAW264.7. (**a**) Summary of the process for collecting LPS-CM. The summary figure was created using BioRender.com. (**b**) RAW264.7 cells were treated with 70COLGA at concentrations of 100 and 200 μg/mL for 2 h and then treated with LPS-CM containing 70COLGA for 30 min. The relative protein levels were analyzed via Western blot analysis.

Pro-inflammatory cytokines are known activators, and we previously confirmed that 70COLGA inhibits STAT activation [38]. Since pro-inflammatory cytokines induce the activation of STAT, we investigated whether 70COLGA directly inhibits STAT activation rather than inhibits the expression of pro-inflammatory cytokines. First, an LPS-conditioned medium (LPS-CM) and the same amount of pro-inflammatory molecule medium, obtained from LPS-induced RAW264.7 cells, was collected and used to treat naïve RAW264.7 cells. Heat-inactivated LPS-CM was used as a negative control. When RAW264.7 cells were treated with LPS-CM, STAT was activated within 30 min, but this effect was suppressed by 70COLGA (Figure 5). These results suggest that 70COLGA not only inhibited the expression of proinflammatory molecules but also directly inhibited the activation of STAT in LPS-induced RAW264.7 cells.

## 4. Discussion

Fermentation is a process by which microorganisms produce useful organic substances [19]. Such bioconversion has recently been applied to the development of new functional materials, such as foods and cosmetics. As safety is the most important issue for use in foods and cosmetics, continuous efforts are needed to reduce the toxicity of various materials [39]. A previous study confirmed that formononetin 7-O-phosphate, a new compound generated through bioconversion technology, improves cell viability and reduces cytotoxicity compared to the existing compound, formononetin [40]. In addition, it was confirmed that *Platycodon grandifloras*-fermented extracts showed an improved cell viability and reduced cytotoxicity compared to existing extracts [41]. In our previous study, we compared and evaluated the anti-inflammatory effects according to the COL extraction method in LPS-induced RAW264.7 cells and confirmed that COL EtOH extracts had excellent anti-inflammatory effects, but they show cytotoxicity at low concentrations [42]. In the present study, we confirmed that 70COLGA alleviated the high cytotoxicity of 70COL using bioconversion technology (Figure 2a). Therefore, 70COLGA may have greater utility if it has a similar anti-inflammatory effect to 70COL.

Inflammation is the body’s response to infection or external stimuli [43]. When macrophages are subjected to external stimuli such as LPS, the NF-κB and MAPK signaling pathways are activated to express COX-2 and iNOS [44,45]. COX-2 is an enzyme that converts arachidonic acid to prostaglandin, and prostaglandin E_2_ (PGE_2_) produced by COX-2 is known to play an important role in the inflammatory process, including fever and pain [46]. If NO, which is produced from L-arginine by iNOS, is continuously produced, it can convert acute inflammation into chronic inflammation, causing inflammatory skin disease and inflammatory joint disease [47,48,49]. Therefore, it is very important to investigate the detailed mechanism of action of various substances to treat inflammatory diseases such as atopic dermatitis and rheumatoid arthritis. In this study, we tried to find the mechanism of action of 70COLGA using an LPS-induced RAW264.7 model. Dexamethasone, a steroid drug, is a synthetic corticosteroid with anti-inflammatory effects and is widely used as an inflammation treatment due to its anti-inflammatory and immunosuppressive effects [50]. However, their continued use causes various side effects such as a high blood pressure, diabetes and hormonal imbalances [51]. Therefore, there is active research on safe natural products with fewer side effects for use as anti-inflammatory treatments. In this study, 70COLGA inhibited the increase in the mRNA and protein levels of iNOS and COX-2 and accordingly reduced NO production in LPS-induced RAW264.7 cells, showing that it has the potential to be used as an anti-inflammatory agent (Figure 2b–f).

The JAK-STAT signaling pathway plays a central role in regulating immune and inflammatory responses [52]. JAK activated by cytokines induces phosphorylation of STAT, and phosphorylated STAT forms homo- or heterodimers and moves to the nucleus, where it induces the expression of inflammation-related genes and regulates inflammation and immune responses [53]. In this study, 70COLGA significantly inhibited the expression of activated STAT1 and 3 in LPS-induced RAW264.7 cells (Figure 3c). The pro-inflammatory cytokine IL-6 is well known to affect the activation of STAT3 directly, and 70COLGA significantly reduced IL-6 mRNA expression in LPS-induced RAW264.7 cells (Figure 3e). In addition, 70COLGA was confirmed to directly inhibit the activation of STAT1 and 3 in LPS-induced RAW264.7 cells (Figure 5b). These results showed that 70COLGA had an anti-inflammatory effect by inhibiting the activation of STAT and regulating the inflammatory response.

Persistent chronic inflammation is associated with an increased risk of cancer and various skin diseases [54,55]. The results presented here indicate that 70COLGA may be used to relieve inflammation and thus prevent cancer and skin diseases. When dermatitis occurs, the moisture supply to the stratum corneum of the epidermis is reduced, resulting in transepidermal water loss (TEWL). Skin barrier disturbance caused by TEWL enables pathogen invasion and causes chronic inflammation, so strengthening the skin barrier is important for inflammatory skin diseases [56]. Additionally, activation of STATs and inflammatory cytokines has been reported to induce the expression of various oncogenes and promote cancer metastasis and growth [57,58]. Therefore, investigating the pharmacological mechanism for skin barrier improvement via 70COLGA will be our future research topic.

## 5. Conclusions

**Figure 6:**
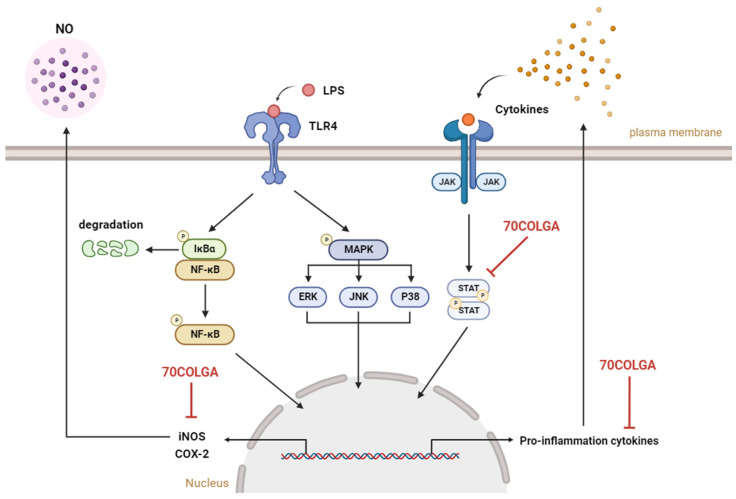
Graphical summary of this study. 70COLGA suppresses pro-inflammatory molecules. NO: nitric oxide, LPS: lipopolysaccharide, TLR4: Toll-like receptor 4, NF-κB: nuclear factor kappa-light-chain-enhancer of activated B cells, IκBα: inhibitory protein kappa B alpha, MAPK: mitogen-activated protein kinase, ERK: extracellular signal-regulated kinase, JNK: jun N-terminal kinase, JAK: janus kinase, STAT: signal transducers and activators of transcription, iNOS: inducible nitric oxide synthase, COX-2: cyclooxygenase 2, 70COLGA: *Chamaecyparis obtusa* (Siebold & Zucc.) Endl. leaf 70% EtOH extract fermented by *Ganoderma applanatum.* This summary figure was created using BioRender.com.

In this study, the anti-inflammatory effects of 70COLGA, a new material generated through bioconversion technology, were investigated. 70COLGA showed an improved cytotoxicity compared to 70COL and inhibited the production of the inflammatory mediators IL-1β, IL-6, IL-27, MIP-2, GM-CSF, G-CSF, and NO and the expression of the pro-inflammatory enzymes COX-2 and iNOS in LPS-induced RAW264.7 cells (Figure 6). In addition, 70COLGA also directly inhibited the activation of STAT1 and 3. 70COLGA has therapeutic potential for the treatment of inflammation.

## Figures and Tables

**Table 1:** Primer sequences used in this study.

Gene Name	Direction	Sequence (5′-3′)
*iNOS* (*mouse*)	Forward	CAGCACAGGAAATGTTTCAGC
	Reverse	TAGCCAGCGTACCGGATGA
*COX-2* (*mouse*)	Forward	TTTGGTCTGGTGCCTGGTC
	Reverse	CTGCTGGTTTGGAATAGTTGCTC
*TNF-α* (*mouse*)	Forward	TATGGCTCAGGGTCCAACTC
	Reverse	CTCCCTTTGCAGAACTCAGG
*IL-1β* (*mouse*)	Forward	TTGACGGACCCCAAAAGATG
	Reverse	AGAAGGTGCTCATGTCCTCA
*IL-6* (*mouse*)	Forward	GGTGACAACCACGGCCTTCCC
	Reverse	AAGCCTCCGACTTGTGAAGTGGT
*GAPDH* (*mouse*)	Forward	GCAAATTCAACGGCACAG
	Reverse	CACCAGTAGACTCCACGAC

## Data Availability

The data used and/or analyzed during the current study are available from the corresponding author upon reasonable request.

## Supplementary Materials

The following supporting information can be downloaded at: https://www.mdpi.com/article/10.3390/pharmaceutics16030365/s1, Table S1: Used reagents in this study; Table S2: Used antibodies in this study; Table S3: Summary of HPLC analysis conditions; Table S4: Peak table of HPLC chromatogram of 70COL; Table S5: Peak table of HPLC chromatogram of 70COLGA.
