# Supplementary material for: Anti-Inflammatory Effects of Chamaecyparis obtusa (Siebold & Zucc.) Endl. Leaf Extract Fermented by Ganoderma applanatum Mycelia
Source: Pharmaceutics. 2024 Mar 5;16(3):365. doi: 10.3390/pharmaceutics16030365 (PMC10974965; doi:10.3390/pharmaceutics16030365)
Supplement: Supplementary file 1 [file pharmaceutics-16-00365-s001.zip › pharmaceutics-2865757-supplementary.pdf]

Anti-Inflammatory Effects of *Chamaecyparis obtusa* (Siebold & Zucc.) Endl. Leaf Extract Fermented by *Ganoderma applanatum* Mycelia

Table S1. Used reagents in this study

**Reagents**	**Catalog number**	**Company**
2,2-Diphenyl-1-picrylhydrazyl (DPPH)	D9132	Sigma Aldrich (St. Louis, MO, USA)
2,2′-Azino-bis(3-ethylbenzothiazoline-6-sulfonic acid) diammonium salt (ABTS)	11557	
Potassium persulfate	216224	
L-Ascorbic acid	A0278	
Tannic acid	403040	
Lipopolysaccharide (LPS)	L3012	
Griess reagent	G4410	
Phenol reagent (Folin-Ciocalteu's reagent)	96703S8130	JUNSEI CHEMICAL CO., LTD (Tokyo, Japan)
Sodium carbonate anhydrous	7541-4405	DAEJUNG CHEMICALS & METALS (Seoul, Republic of Korea)
3-(4,5-dimethylthiazol-2-Yl)-2,5-diphenyltetrazolium bromide (MTT)	M1415	Duchefa Biochemie (Haarlem, Netherlands)

Table S2. Used antibodies in this study

Antibody	Catalog number	Company
COX-2	#12282	Cell Signaling Technology (Danvers, MA, USA)
pT/Y-p44/42 MAPK (Erk1/2)	#9101	
p44/42 MAPK (Erk1/2)	#4695	
pT/Y-SAPK/JNK	#9251	
SAPK/JNK	#9252	
pT/Y-p38 MAPK	#9211	
p38 MAPK	#9212	
pS-IκBα	#2859	
pS-NFκB	#3033	
pY-STAT1	#8826	
STAT1	#9172	
pY-STAT3	#9145	
STAT3	#30835	
IκBα	sc-1643	Santa Cruz Biotechnology (Santa Cruz, CA, USA)
NFκB	sc-8008	
β-Actin	sc-47778	
iNOS	PA-1036	Invitrogen (Waltham, MA, USA)
HRP-tagged anti-rabbit	A21010	Abbkine (Wuhan, China)
HRP-tagged anti-mouse	ADI-SAB-100	Enzo Life Science (Farmingdale, NY, USA)

Table S3. Summary of HPLC analysis conditions

**Instrument**	Agilent HPLC 1260		
**Parameter**	Condition		
**Column**	Agilent Eclipse XDB-C18 (5 μm, 4.6 × 250 mm)		
**Column temp.**	30 ℃		
**Injection vol.**	15 μl		
**Flow rate**	1 ml/min		
**Wavelength**	Amentoflavone	330 nm	
	Quercitrin, Myricetin	376 nm	
**Mobile phase A**	DIW		
**Mobile phase B**	ACN		
**Gradient**	**Time (min)**	**A (%)**	**B (%)**
	initial	80	20
	5.0	65	35
	8.0	65	35
	23.0	50	50
	23.1	0	100
	28.0	0	100
	28.1	80	20
	33.0	80	20

Table S4. Peak table of HPLC chromatogram of 70COL. (**a**) Quercitrin. (**b**) Amentoflavone.

(a) 70COL – Wavelength 330 nm

#	Time	Area	Height	Width	Area%	Symmetry	Name
1	1.038	3.70E-01	1.00E-01	0.0534	0.026	1.25	
2	2.064	56.1	10.3	0.0796	3.893	1.048	
3	2.22	64.2	13	0.0696	4.456	2.095	
4	2.299	74	15.2	0.0768	5.133	0.994	
5	2.476	3.8	5.70E-01	0.0932	0.263	0.329	
6	2.891	42.3	12.4	0.0518	2.94	0.789	
7	3.031	85.9	16.9	0.0793	5.962	0.934	
8	3.227	34.1	3.7	0.1378	2.367	0.915	
9	3.559	1.1	2.00E-01	0.089	0.076	0.988	
10	3.769	7.1	9.20E-01	0.1207	0.496	0.686	
11	4.188	23	3.2	0.1053	1.595	0.791	
12	4.527	8.4	9.30E-01	0.1274	0.58	1.151	
13	4.89	132.8	17.8	0.111	9.219	0.78	
14	5.205	1.4	3.10E-01	0.068	0.097	1.176	
15	5.334	1.20E-01	4.20E-02	0.0483	0.008	1.643	
16	5.504	5.7	6.60E-01	0.1415	0.396	0.976	
17	5.758	5.2	8.40E-01	0.1	0.36	2.757	
18	5.884	22.6	3.6	0.0949	1.571	1.027	
19	6.089	5.5	1	0.0854	0.383	0.674	
20	6.371	217.3	28.9	0.1096	15.081	0.584	Quercitrin
21	6.659	24.7	3.3	0.1101	1.717	0.984	
22	6.876	7	1.5	0.0708	0.484	1.084	
23	7.004	27.3	3.6	0.1195	1.898	0.868	
24	7.325	14.8	2.2	0.1038	1.03	0.576	
25	7.882	5.1	4.50E-01	0.1587	0.351	0.697	
26	8.265	29.3	3.2	0.1322	2.033	1.036	
27	8.852	3.1	5.10E-01	0.0961	0.217	0.879	
28	9.151	6.40E-01	9.70E-02	0.1046	0.044	0.555	
29	9.674	3.6	2.90E-01	0.1839	0.253	0.798	
30	10.013	3.7	2.60E-01	0.1954	0.26	0.605	
31	10.642	1.8	1.70E-01	0.1596	0.128	0.778	
32	10.914	2.7	1.70E-01	0.2059	0.187	0.476	
33	12.558	3.40E-01	3.60E-02	0.121	0.024	3.227	
34	12.882	6.5	5.60E-01	0.1746	0.452	0.884	
35	13.078	6.1	4.90E-01	0.1918	0.425	0.486	
36	13.784	5	2.90E-01	0.2302	0.347	0.601	
37	14.644	16.9	1.4	0.1854	1.174	0.714	
38	15.575	360.4	17.1	0.342	25.015	1.101	
39	17.238	3.3	1.90E-01	0.2383	0.229	1.988	
40	17.624	83	6.1	0.2039	5.763	0.654	
41	20.623	26.4	1.6	0.2373	1.836	0.635	
42	22.185	3.2	3.00E-01	0.1588	0.225	1.063	
43	22.497	14.5	7.20E-01	0.2752	1.003	0.626	

(b) 70COL – Wavelength 376 nm

#	Time	Area	Height	Width	Area%	Symmetry	Name
1	1.041	4.00E-01	9.60E-02	0.0611	0.078	1.109	
2	2.059	6.7	1	0.0941	1.302	1.27	
3	2.222	20.9	4.1	0.069	4.039	2.013	
4	2.3	20.3	4.4	0.0743	3.934	1.024	
5	2.479	10.8	7.40E-01	0.188	2.093	0.237	
6	2.898	11.6	3.5	0.0506	2.238	0.831	
7	3.032	44.6	8	0.0811	8.625	0.685	
8	3.373	6.2	4.10E-01	0.2024	1.197	3.032	
9	4.192	1.3	1.80E-01	0.1068	0.253	0.827	
10	4.531	4.20E-01	6.90E-02	0.0908	0.081	1.971	
11	4.893	6.4	9.10E-01	0.1044	1.239	0.874	
12	5.008	3.1	4.90E-01	0.0947	0.592	0.379	
13	5.207	1.8	3.30E-01	0.0814	0.349	0.996	
14	5.468	7.1	7.60E-01	0.1306	1.364	1.283	
15	5.893	10.7	1.6	0.1007	2.074	0.875	
16	6.094	4.8	7.50E-01	0.0983	0.927	0.844	
17	6.371	140.1	19.3	0.1087	27.093	0.609	
18	6.752	2.2	4.10E-01	0.0771	0.419	0.934	
19	6.874	5.5	7.60E-01	0.1036	1.057	0.688	
20	7.201	4.10E-01	1.10E-01	0.0565	0.079	1.363	
21	7.324	6.8	8.80E-01	0.1122	1.317	0.59	
22	8.268	7.9	1	0.1162	1.537	0.787	
23	8.852	1.1	1.90E-01	0.0934	0.218	0.894	
24	9.142	3.20E-01	4.00E-02	0.1004	0.062	0.389	
25	9.684	8.20E-01	8.20E-02	0.1376	0.159	1.759	
26	10.014	5.5	4.10E-01	0.1942	1.07	0.514	
27	10.643	3.00E-01	3.60E-02	0.1114	0.058	2.698	
28	12.859	2.2	1.30E-01	0.2184	0.426	0.318	
29	13.837	1.3	9.50E-02	0.1755	0.259	0.769	
30	14.648	3.2	2.60E-01	0.1807	0.617	0.699	
31	15.574	137.8	6.4	0.3474	26.656	1.036	Amentoflavone
32	17.623	30.4	2.2	0.2037	5.879	0.656	
33	20.626	7.3	4.70E-01	0.2245	1.416	0.524	
34	22.188	1.3	1.10E-01	0.1538	0.243	1.029	
35	22.473	5.4	2.50E-01	0.2675	1.051	0.369	

Table S5. Peak table of HPLC chromatogram of 70COLGA. (**a**) Quercitrin. (**b**) Amentoflavone.

(a) 70COLGA– Wavelength 330 nm

#	Time	Area	Height	Width	Area%	Symmetry	Name
1	0.455	6.30E-02	2.10E-02	0.0499	0.013	1.141	
2	2.068	14.1	2	0.0999	2.922	1.714	
3	2.221	29.5	3.6	0.1236	6.1	1.154	
4	2.309	9.6	3.1	0.0521	1.986	0.496	
5	2.482	10.7	6.30E-01	0.2143	2.211	0.214	
6	2.895	20.4	6.1	0.053	4.232	0.79	
7	3.036	63.4	10.1	0.0889	13.114	0.573	
8	3.396	4.4	5.40E-01	0.1219	0.916	0.711	
9	4.192	4.5	4.60E-01	0.1392	0.94	1.449	
10	4.548	1.2	9.80E-02	0.163	0.242	1.783	
11	4.886	19.3	2.2	0.126	3.997	0.596	
12	5.202	7.50E-01	1.90E-01	0.063	0.156	0.933	
13	5.332	2.7	4.80E-01	0.0878	0.557	1.031	
14	5.473	7.1	9.00E-01	0.1137	1.467	0.707	
15	5.73	9.70E-01	1.60E-01	0.0935	0.201	0.84	
16	5.89	2.9	4.90E-01	0.0912	0.598	1.085	
17	6.093	3.9	6.00E-01	0.1044	0.811	0.778	
18	6.372	209.9	30.5	0.1042	43.454	0.727	
19	6.775	2.4	4.00E-01	0.087	0.502	1.878	
20	6.874	3.2	5.50E-01	0.0876	0.658	0.889	
21	7.2	3.20E-01	9.60E-02	0.0525	0.065	1.122	
22	7.325	7.4	9.40E-01	0.1157	1.528	0.591	
23	8.272	5.8	7.40E-01	0.1157	1.205	0.64	
24	8.847	1.1	6.80E-02	0.2091	0.231	0.168	
25	9.64	7.90E-01	6.50E-02	0.149	0.164	0.741	
26	10.004	1.3	1.10E-01	0.1539	0.276	0.387	
27	12.921	1.3	8.80E-02	0.1818	0.261	1.552	
28	13.946	5.80E-01	5.10E-02	0.1425	0.119	2.801	
29	14.68	2.4	1.60E-01	0.1813	0.495	0.575	
30	15.571	42.7	2	0.3383	8.841	0.872	Amentoflavone
31	17.655	4.3	3.00E-01	0.1972	0.89	0.636	
32	20.648	4.1	2.50E-01	0.2086	0.849	0.408	

(b) 70COLGA – Wavelength 376 nm

#	Time	Area	Height	Width	Area%	Symmetry	Name
1	2.086	76.4	11.8	0.0997	4.894	1.528	
2	2.216	81.5	13.3	0.0896	5.221	1.453	
3	2.304	66.5	12.5	0.0762	4.264	0.987	
4	2.485	5.1	4.40E-01	0.1556	0.326	9.53E-02	
5	2.892	56.9	16.7	0.0517	3.645	0.812	
6	3.034	109.1	21.3	0.0777	6.994	0.901	
7	3.225	32.3	3.1	0.1525	2.073	0.762	
8	3.734	1.4	1.90E-01	0.1128	0.09	0.766	
9	4.19	54.7	7.7	0.1068	3.506	0.806	
10	4.476	12.6	1.2	0.1416	0.807	0.642	
11	4.885	351	44.2	0.1167	22.49	0.68	
12	5.328	1.2	3.10E-01	0.0642	0.074	1.046	
13	5.539	32.2	4.7	0.102	2.065	1.025	
14	5.719	27.1	3.9	0.1007	1.739	0.799	
15	5.88	31.6	5.1	0.0919	2.024	1.036	
16	6.078	4.8	9.30E-01	0.085	0.31	0.729	
17	6.372	320.2	45.9	0.1053	20.519	0.717	Quercitrin
18	6.648	15.3	1.9	0.1153	0.978	0.657	
19	7.009	75	7.3	0.1499	4.807	1.158	
20	7.326	18.9	2.4	0.114	1.21	0.613	
21	8.271	17.5	2.1	0.1215	1.12	0.754	
22	8.854	1.2	1.80E-01	0.105	0.076	0.752	
23	9.663	2.2	1.90E-01	0.1694	0.14	0.802	
24	10.637	1	1.20E-01	0.1376	0.065	0.882	
25	12.904	5.3	3.20E-01	0.2218	0.342	0.642	
26	13.93	2	1.40E-01	0.1913	0.131	1.647	
27	14.671	11.1	8.00E-01	0.203	0.709	0.7	
28	15.575	116.2	5.4	0.3434	7.448	0.893	
29	17.653	12.1	8.30E-01	0.2142	0.772	0.618	
30	20.66	13	8.40E-01	0.2304	0.833	0.578	
31	22.478	5.1	2.10E-01	0.3025	0.328	1.575	
